# Computed Tomography–Based Stiffness Measures of Trabecular Bone Microstructure: Cadaveric Validation and In Vivo Application

**DOI:** 10.1002/jbm4.10627

**Published:** 2022-05-05

**Authors:** Indranil Guha, Xialiou Zhang, Chamith S. Rajapakse, Elena M. Letuchy, Gregory Chang, Kathleen F. Janz, James C. Torner, Steven M. Levy, Punam K. Saha

**Affiliations:** ^1^ Department of Electrical and Computer Engineering University of Iowa Iowa City IA USA; ^2^ Departments of Radiology and Orthopedic Surgery University of Pennsylvania Philadelphia PA USA; ^3^ Department of Epidemiology University of Iowa Iowa City IA USA; ^4^ Department of Radiology New York University Grossman School of Medicine New York NY USA; ^5^ Department of Health and Human Physiology University of Iowa Iowa City IA USA; ^6^ Department of Preventive and Community Dentistry University of Iowa Iowa City IA USA; ^7^ Department of Radiology University of Iowa Iowa City IA USA

**Keywords:** ANSYS SOFTWARE, ASH DENSITY, BODY SIZE AND COMPOSITION, COMPRESSIVE AND SHEAR LOADING, CT IMAGING, MICROSTRUCTURE, NONLINEAR FEA, OSTEOPOROSIS, TB STIFFNESS, TRABECULAR BONE, VON MISES STRESS, YOUNG'S MODULUS

## Abstract

Osteoporosis causes bone fragility and elevates fracture risk. Applications of finite element (FE) analysis (FEA) for assessment of trabecular bone (Tb) microstructural strength at whole‐body computed tomography (CT) imaging are limited due to challenges with Tb microstructural segmentation. We present a nonlinear FEA method for distal tibia CT scans evading binary segmentation of Tb microstructure, while accounting for bone microstructural distribution. First, the tibial axis in a CT scan was aligned with the FE loading axis. FE cubic mesh elements were modeled using image voxels, and CT intensity values were calibrated to ash density defining mechanical properties at individual elements. For FEA of an upright volume of interest (VOI), the bottom surface was fixed, and a constant displacement was applied at each vertex on the top surface simulating different loading conditions. The method was implemented and optimized using the ANSYS software. CT‐derived computational modulus values were repeat scan reproducible (intraclass correlation coefficient [ICC] ≥ 0.97) and highly correlated (*r* ≥ 0.86) with the micro‐CT (μCT)‐derived values. FEA‐derived von Mises stresses over the segmented Tb microregion were significantly higher (*p* < 1 × 10^−11^) than that over the marrow space. In vivo results showed that both shear and compressive modulus for males were higher (*p* < 0.01) than for females. Effect sizes for different modulus measures between males and females were moderate‐to‐high (≥0.55) and reduced to small‐to‐negligible (<0.40) when adjusted for pure lean mass. Among body size and composition attributes, pure lean mass and height showed highest (*r* ∈ [0.45 0.56]) and lowest (*r* ∈ [0.25 0.39]) linear correlation, respectively, with FE‐derived modulus measures. In summary, CT‐based nonlinear FEA provides an effective surrogate measure of Tb microstructural stiffness, and the relaxation of binary segmentation will extend the scope for FEA in human studies using in vivo imaging at relatively low‐resolution. © 2022 The Authors. *JBMR Plus* published by Wiley Periodicals LLC on behalf of American Society for Bone and Mineral Research.

## Introduction

Osteoporosis is a bone‐related disease prevalent among elderly people and associated with reduced bone mineral density (BMD), bone quality, and bone strength, which results in an increased fracture risk.^(^ ^1^ ^)^ Nearly one in two women and one in four men suffer at least one osteoporotic fragility fracture in their lifetimes.^(^ ^2^ ^)^ BMD computed from dual‐energy X‐ray absorptiometry (DXA) is the current clinical standard for definition and diagnosis of osteoporosis.^(^ ^3^ ^)^ It has been shown that BMD explains only 60% to 70% of the variability in bone strength and fracture risk,^(^ ^4^ ^)^ and the remaining variability is attributed to the collective contributions of several other factors, including the quality of trabecular bone (Tb) microstructure and its impact on strength.^(^ ^5^, ^6^, ^7^, ^8^, ^9^, ^10^ ^)^ It has also been demonstrated that bone quality and strength vary with sex, age, and body size and composition parameters, including height, weight, pure lean mass, and fat mass.^(^ ^11^, ^12^, ^13^, ^14^, ^15^, ^16^, ^17^ ^)^

Finite element (FE) analysis (FEA) has been widely applied to compute Tb strength and assessment of fracture risk using high‐resolution imaging.^(^ ^9^, ^18^, ^19^, ^20^, ^21^, ^22^ ^)^ Micro–computed tomography (μCT)‐based FEA is widely used as a reference method for computation of Tb strength, and several studies have convincingly demonstrated that FEA‐based Tb strength measures from μCT scans highly correlate with values observed in mechanical experiments.^(^ ^9^, ^19^, ^20^, ^23^ ^)^ FEA methods have been applied on high‐resolution peripheral quantitative CT (HR‐pQCT) and magnetic resonance imaging (MRI) for in vivo assessment of Tb strength.^(^ ^18^, ^24^, ^25^, ^26^, ^27^, ^28^ ^)^

Recently, emerging whole‐body CT, commonly available in a clinical setting, has drawn interest as a viable imaging modality for in vivo assessment of bone microstructural quality and strength at peripheral sites. Also, CT imaging overcomes several major challenges of MRI and HR‐pQCT techniques, related to slow scanning, limited field‐of‐view, and failure to provide quantitative BMD (g/cc) (in the case of MRI).^(^ ^29^ ^)^ Although the high‐resolution features of CT scanners allow visualization of Tb microstructures, the spatial resolution offered by the state‐of‐the‐art technologies is comparable or nominally higher than the human Tb thickness and falls in the range where computed bone microstructural measures are sensitive to resolution differences among scanners. Low spatial resolution results in partial volume errors causing smaller trabeculae to be artifactually broken or missing following segmentation. This makes FEA of Tb from CT images challenging as degenerated connectivity leads to disruption in FEA stress flow along the Tb network and the mechanical contributions of subsequent elements are ignored, contributing to cumulative errors. Although BMD‐adjusted nonlinear FEA on CT images was adopted in several studies, they were applied mostly at central sites, particularly at the hip, and did not account for the mechanical properties at the scale of bone microstructure.^(^ ^30^, ^31^ ^)^ In this work, we develop and validate a nonlinear FEA method for CT‐based computation of Tb stiffness in terms of Young’ modulus or simply modulus without requiring segmentation of Tb microstructure, while characterizing mechanical stress‐strain behavior at the microstructural level. Tb modulus was computed under compressive and shear loading conditions and reproducibility and accuracy of the computed modulus measures were examined in a postmortem study using cadaveric ankle specimens, repeat CT scans, and μCT imaging. The method was also applied on in vivo human distal tibia CT scans from males and females, normative statistics of different Tb modulus measures were derived, and the associations of modulus measures with sex, body size, and body composition metrics were examined.

## Materials and Methods

Our methods and experiments were designed to optimize and evaluate compressive and shear modulus computation algorithms for Tb microstructure using BMD‐adjusted nonlinear FEA and to apply the methods on in vivo human scans. The following materials and methods were used in our experiments: (i) CT imaging, (ii) μCT imaging, (iii) cadaveric ankle specimens, (iv) human subjects, (v) image preprocessing, (vi) nonlinear FE modeling and analysis, (vii) optimization of nonlinear FEA parameters, and (viii) experiments and data analysis.

### CT imaging

CT scans of the distal tibia at high‐resolution mode were collected on a 128 slice Siemens SOMATOM Definition Flash scanner (Siemens, Forchheim, Germany) at the University of Iowa Comprehensive Lung Imaging Center. The scans were acquired in single tube spiral acquisition mode using the following parameters: 120 kV; 200 mA effective; 1 second rotation speed; pitch factor 1.0; detector rows 16; scan time 23.2 seconds; collimation 16 × 0.6 mm; total effective dose equivalent 170 μSv ≈ 20 days of environmental radiation in the United States. Siemens z‐UHR scan mode was applied enabling Siemens dual z sampling technology, which splits the signal on 0.6‐mm detectors delivering 0.3 mm effective spatial resolution in the z‐direction.^(^ ^29^ ^)^ Images were reconstructed at 300 μm thickness and 200 μm slice‐spacing using a normal cone beam method with a special U70u kernel achieving high spatial resolution. Three repeat CT scans were collected for each specimen on the same day after repositioning the specimen on the scanner table between scans. A Gammex RMI 467 Tissue Characterization Phantom (Gammex RMI, Middleton, WI, USA) was scanned on each day of a tibia scan using the same protocol to calibrate CT numbers into BMD.

### μCT imaging

μCT imaging of distal tibia specimens was performed on a Micro‐cat II (Siemens Pre‐Clinical, Knoxville, TN, USA) cone beam scanner. The following parameters were used for μCT scans: 100 kV, 200 mAs, 720 projections over 220 degrees, exposure of 1 second per projection, scan time: 12 minutes, 2 mm Al filter for beam hardening; scan‐length 29.5 mm; and field of view (FOV) on the xy plane 44.2 × 44.2 mm^2^. μCT scans were reconstructed at 28.8 μm isotropic voxel size with 1024 slices and image array of 1536 × 1536 using filtered back‐projection.

### Cadaveric ankle specimens

Twenty‐four fresh frozen cadaveric ankle specimens were collected from 17 body donors (age, mean ± standard deviation [SD]: 79.60 ± 13.20 years; nine females) under the Deeded Bodies Program at The University of Iowa. Specimens from donors who had a bone tumor, metastasis, or fracture at the tibia were excluded. These specimens were separated at mid‐tibia with the ankle joint, foot, and soft tissues preserved and kept frozen in sealed bags. The specimens were thawed at room temperature before any imaging or specimen preparation step and kept frozen in between these steps. First, distal tibia CT scans of these specimens were acquired. To prepare for μCT scans, soft tissues were removed and the tibia was separated from the ankle joint to fit into the scanner then wrapped with wet gauze.

### Human subjects

Fifty healthy males and fifty healthy females (age, range 19 to 20 years; mean ± SD: 19.40 ± 0.40 years) were randomly selected from the Iowa Bone Development Study (IBDS).^(^ ^32^, ^33^ ^)^ High‐resolution CT scans of distal tibia were acquired for these subjects under the IBDS study design using the same imaging protocol described in CT Imaging. These high‐resolution CT images were used in our experiments, together with the sex, height, weight, and DXA‐derived lean body mass measures, ie, whole‐body fat‐free mass minus bone mineral content, collected under the parent study. The IBDS study was approved by the University of Iowa Institutional Review Board, and the use of the data under the current study is Health Insurance Portability and Accountability Act (HIPAA)‐compliant with written informed consent collected from all participants. This study population has been reported in different research contexts.^(^ ^34^ ^)^

### Image preprocessing

An image processing cascade was designed for CT scans to: (i) align the tibial bone axis with the image z‐ and x‐axis along the tibiofemoral direction and interpolate the image at 150 μm isotropic voxel size, (ii) compute ash density from the calcium hydroxyapatite (CHA) (g/cc) density using calibration phantom scans, and (iii) select the volume of interest (VOI) using anatomic reference. To minimize sampling‐induced loss of spatial resolution, bone alignment and isotropic voxel interpolation steps were combined, requiring a single step of image sampling.

#### Bone alignment

Tibia scans were aligned to the coordinate axes using two separately computed transformation metrics. The first transformation was to align the tibial bone axis with the image z‐axis. For this purpose, the tibial bone region was segmented on CT images using a previously validated automated algorithm,^(^ ^35^ ^)^ and the tibial bone axis was computed over the 40% peeled bone region proximal to the 8% distal site. The second transformation representing an in‐plane rotation was computed to align the image x‐axis to the tibiofemoral direction—a unit vector from the center of gravity (C.G.) of the tibia toward the C.G. of the fibula. Image x‐axis was aligned to the tibiofemoral direction to attribute an anatomic reference to x‐ and y‐shear loading directions. These two transformation metrics were combined with 150 μm isotropic voxel interpolation using the windowed‐sync method.^(^ ^36^ ^)^

#### CHA and ash density computation

First, CT numbers in Hounsfield units (HU) were converted to CHA density in two conversion steps: (i) CT HU numbers to BMD (g/cc) and (ii) BMD to CHA density. CT HU numbers were calibrated to BMD values using the calibration phantom scan described in CT Imaging and a previously‐reported fully automated method.^(^ ^29^ ^)^ At a voxel p, the CHA density $\rho_{\mathrm{CHA}}\left(p\right)$ was obtained from its CT‐derived BMD value $\rho_{\mathrm{BMD}}\left(p\right)$ using known densities $D_{\mathrm{CHA}}$ (3.18 g/cc) and $D_{\text{water}}$ (1 g/cc) of CHA and marrow, respectively, and the following linear equation:

(1) $$\rho_{\mathrm{CHA}}\left(p\right)=\frac{\left(\rho_{\mathrm{BMD}}\left(p\right)-D_{\text{water}}\right)}{\left(D_{\mathrm{CHA}}-D_{\text{water}}\right)}\times D_{\mathrm{CHA}}$$

Specifically, the above equation determines the fractional occupancy of CHA in a CHA‐water mixture model that results to the observed BMD value of $\rho_{\mathrm{BMD}}\left(p\right)$ at voxel p and then multiplies the fractional occupancy of CHA by the CHA material density $D_{\mathrm{CHA}}$. Finally, the ash density $\rho_{\mathrm{ash}}\left(p\right)$ at the voxel p was computed using Equ. (2)^(^ ^21^, ^22^ ^)^:

(2) $$\rho_{\mathrm{ash}}\left(p\right)=0.0633+0.887\rho_{\mathrm{CHA}}\left(p\right)$$

#### VOI selection

**Fig. 1:**
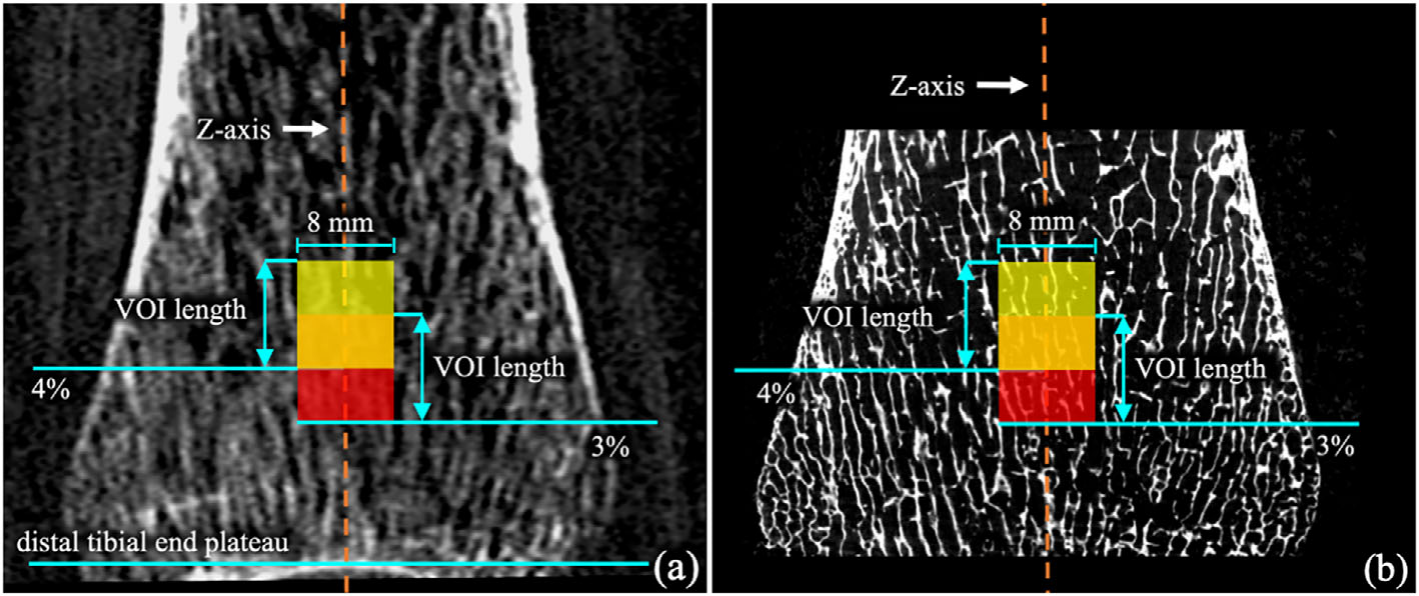
VOI location for FEA on a sagittal slice from registered CT (*A*) and μCT (*B*) scans of a cadaveric ankle specimen.

**Fig. 2:**
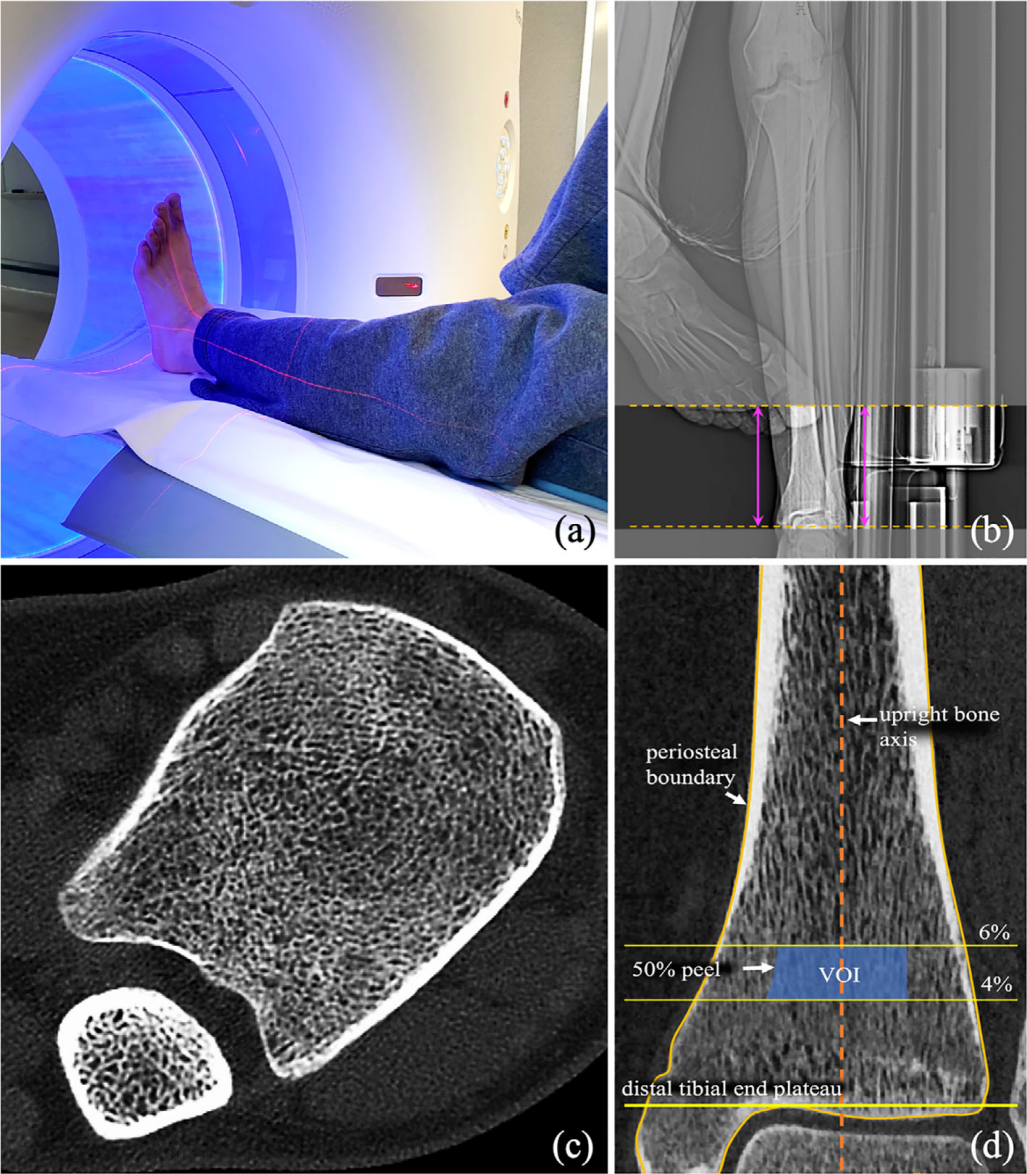
Setup for in vivo human ankle scan on a Siemens Flash CT scanner and VOI selection for FEA. (*A*) Positioning of the ankle after visually aligning the tibial axis with scanner center using laser rays to achieve maximum image resolution. (*B*) Position of the FOV on an anterior‐posterior projection CT scout scan including the distal tibial end plateau used to determine the VOI location for FEA. (*C*) An axial image slice from the reconstructed CT scan. (*D*) Location of the VOI on a sagittal image slice for FEA. FOV = field of view.

**Fig. 3:**
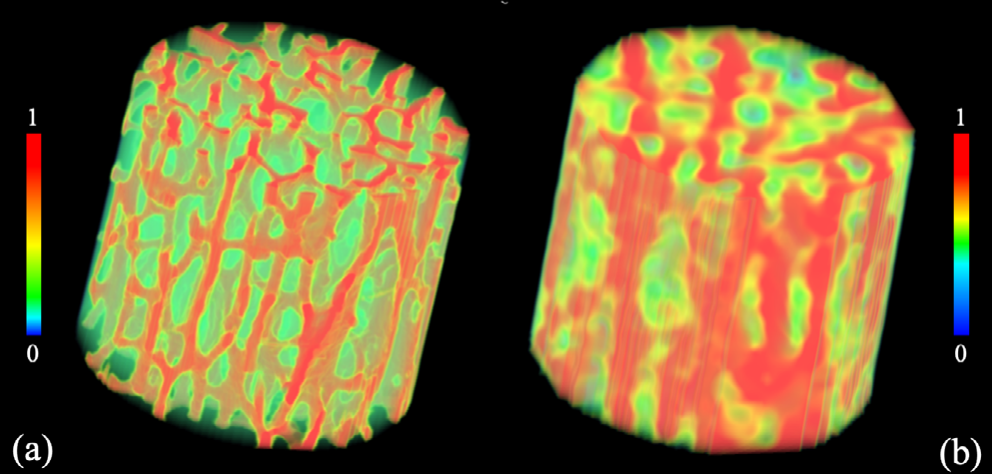
Color‐coded volume rendition of matching VOIs from μCT (*A*) and CT (*B*) images after normalizing the intensity between 0 and 1. Due to different intensity distributions in μCT and CT images, different color‐scales and transparency were used to highlight the Tb microstructures.

Upright axial VOIs were selected from CT and μCT images after aligning the tibia axis with the image z‐axis. Fig. 1 describes the VOI selection process for the cadaveric CT versus μCT experiments. First, a fully automated algorithm^(^ ^35^ ^)^ was used to retrospectively locate the reference axial plane at the distal tibial end plateau after upright alignment of the distal tibial bone axis (see Fig. 1 *A*). Next, cylindrical VOIs of 6.75 mm in length (~2% of average tibial length) and 8 mm in diameter covering 3%–5% and 4%–6% of tibial regions proximal to the distal tibial end plateau were selected for FEA. μCT scans (*n* = 13) were registered to corresponding aligned CT scans using ITK‐SNAP software (Kitware Inc., Carrboro, NC, USA), while preserving its original isotropic voxel resolution, to select the matching μCT VOIs for FEA (see Fig. 1 *B*). For three specimens, their μCT scans did not include 6% tibial region; therefore, only one μCT VOI representing 3%–5% tibial region was used for those specimens. Thus, a total of 23 pairs of matching VOIs from CT and μCT scans were used for comparing CT‐based and μCT‐based FEA measures. Fig. 2 shows the setup for in vivo ankle CT imaging (Fig. 2*A*) along with the FOV position on a scout scan (Fig. 2*B*) and a reconstructed axial image slice (Fig. 2*C*). The protocol for VOI location for in vivo experiments is illustrated in Fig. 2*D*. Similar to the cadaveric experiment, first, the reference axial plane is located at the distal tibia end plateau after upright alignment of the distal tibial bone axis. Then a quasi‐cylindrical VOI was selected over 4%–6% tibial length of the 50% peeled bone region; 50% axial peel was computed using in‐plane distance transform^(^ ^37^, ^38^ ^)^ from the periosteal boundary of the cortical bone and then thresholding at 50% of the highest distance transform values on individual axial slices. VOI dimensions were chosen so that their cross‐sectional diameters are always greater than their lengths to minimize the microstructural disintegrity effects near the lateral surface.^(^ ^39^ ^)^ Volume rendition of matching VOIs from μCT and CT images are shown in Fig. 3*A,B*.

### Nonlinear FE modeling and analysis

The nonlinear FEA method was implemented within the computational platform of ANSYS software (ANSYS Mechanical 2019 R2, Ansys Inc., Southpointe, PA, USA). FE mesh was directly constructed from the isotropic image voxel grid after aligning the tibial bone axis to the upright position. Specifically, each voxel was modeled as a cubical mesh element with its center at the voxel coordinate location. Each of these mesh elements consisted of 12 edge and eight vertex elements corresponding to the voxel edges and vertices, and the connectivity between two adjacent mesh elements was defined by specifying their common edges and vertices. Each mesh element was assigned a nonlinear isotropic mechanical property based on the ash density at the corresponding voxel.

**Fig. 4:**
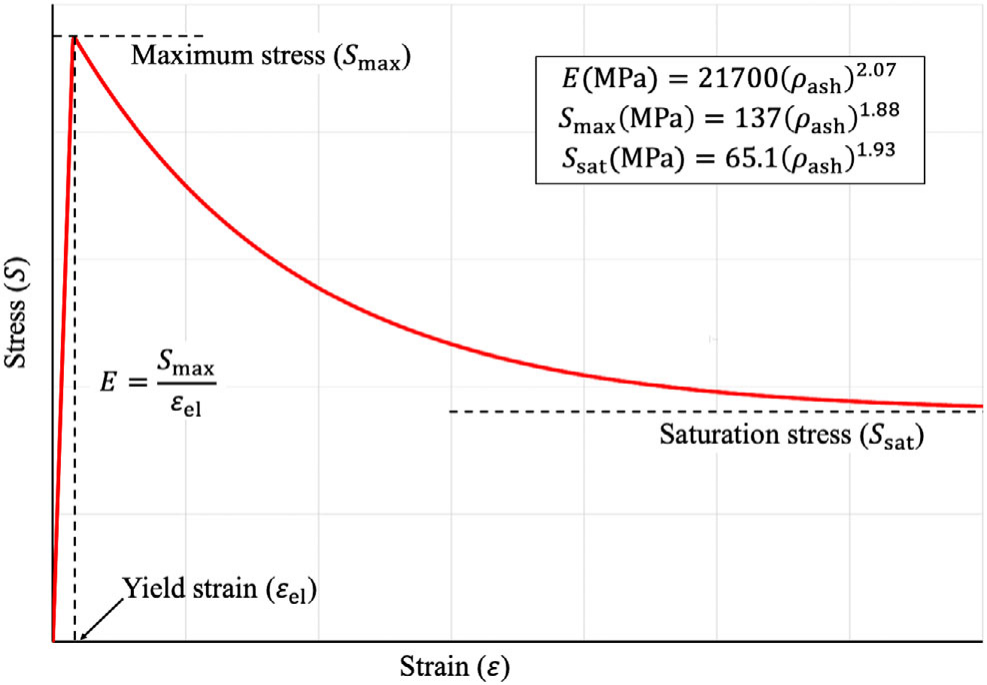
Nonlinear stress‐strain relationship for FEA. The relationship in the plastic phase, beyond the yield strain $\varepsilon_{\mathrm{el}}$, was modeled using the Voce isotropic hardening law.^(^ ^41^ ^)^

The nonlinear stress‐strain relationship defining the material behavior of each mesh element was divided into two segments characterizing their elastic and plastic behavior (Fig. 4), respectively. The nonlinear stress‐strain model was adopted from a previously validated model by Keyak and colleagues^(^ ^40^ ^)^ except that the stress‐strain relationships of both failure and saturation phases within the plastic region were combined into one exponential function. Let $p_{e}$ denote the mesh element corresponding to an image voxel p. The elastic modulus $E\left(p_{e}\right)$ (MPa), maximum stress $S_{\max}\left(p_{e}\right)$ (MPa), and saturation stress $S_{\mathrm{sat}}\left(p_{e}\right)$ (MPa) at the element $p_{e}$ are defined using $\rho_{\mathrm{ash}}\left(p\right)$ as follows^(^ ^40^ ^)^:

(3) $$E\left(p_{e}\right)=21700{\left(\rho_{\mathrm{ash}}\left(p\right)\right)}^{2.07}$$

(4) $$S_{\max}\left(p_{e}\right)=137{\left(\rho_{\mathrm{ash}}\left(p\right)\right)}^{1.88}$$

(5) $$S_{\mathrm{sat}}\left(p_{e}\right)=65.1{\left(\rho_{\mathrm{ash}}\right)}^{1.93}$$

At a mesh element $p_{e}$, the stress‐strain relationship $S_{p_{e}}\left(\varepsilon \right)$ | $\varepsilon >\varepsilon_{\mathrm{el}}\left(p_{e}\right)$, depicting a strain softening behavior in the plastic region beyond the yield strain $\varepsilon_{\mathrm{el}}\left(p_{e}\right)$, was modeled using the Voce isotropic hardening law^(^ ^41^ ^)^ derived from mixed Swift‐Voce isotropic hardening equation^(^ ^42^ ^)^ (Equ. 6) after setting the value of $R_{2}$ to 0.

(6) $$S_{p_{e}}\left(\varepsilon \right)=S_{\max}\left(p_{e}\right)+R_{1}*\left(1-e^{-b\left(\varepsilon -\varepsilon_{\mathrm{el}}\left(p_{e}\right)\right)}\right)+R_{2}*\left(\varepsilon -\varepsilon_{\mathrm{el}}\left(p_{e}\right)\right)\;|\;\varepsilon >\varepsilon_{\mathrm{el}}\left(p_{e}\right)$$

A negative value of $R_{1}$ was used in the above equation to model a material property with negative stress‐strain behaviors beyond the yield strain, which is the sole option within the ANSYS platform for simulating a plastic region with a negative stress‐strain slope. The parameters of the Voce isotropic hardening equation were optimized to resemble with the previously validated stress‐strain curve by Keyak and colleagues,^(^ ^40^ ^)^ and the following parameter values were used: $R_{1}=S_{\mathrm{sat}}\left(p_{e}\right)-S_{\max}\left(p_{e}\right)$ and b=10. Finally, a Poisson's ratio of 0.3 was assigned to each mesh element and boundary conditions were imposed to simulate a physical mechanical experiment of axial compressive and shear loading. The bottom surface of the VOI was fixed in all three coordinate directions and a fixed displacement was applied along the x‐, y‐, or z‐axes on the top surface nodes while restricting their movements in the other two directions. Loading conditions of compressive mechanical test along the tibial bone axis were simulated by applying a displacement along the image z‐axis. Mechanical experiments of shear loading along the bone cross‐sectional plane was simulated by applying a displacement along the image x‐ or y‐axis.

All FE analysis were performed on a Linux machine equipped with 64 GB RAM, 72 cores Intel(R) Xeon(R) Gold 6240 CPU at 2.60 GHz processor, and four Tesla V100‐SXM2 GPUs with 32 GB memory each. Cubic mesh elements were modeled using 3D 8‐Node structural solid (SOLID185) provided by the ANSYS software. Under the boundary conditions mentioned earlier, a total displacement equivalent to 1% of the VOI length in each direction was uniformly distributed over a predefined number of substeps. Newton‐Raphson's method was used for iterative convergence of force and displacement during each sub‐step. The ANSYS feature of NLGEOM was turned on to account for changes in stiffness properties of individual surfaces and edges of mesh elements during the nonlinear shape deformation. Finally, Young's modulus for axial and shear loading were computed as the ratio of average von Mises stress (MPa)^(^ ^42^ ^)^ of volume elements on the VOI top surface and applied displacement. In this work, Young's modulus computed from compressive axial loading along the z‐axis and shear loading along the x‐axis (or y‐axis) is referred to as compressive and x (or y) shear modulus, respectively. It may be noted that stiffness (N/m) of an object with a specified geometry is directly linked to the modulus (MPa) of the object material.

Reference bone modulus measures using compressive as well as shear loading were computed from ex vivo μCT scans of cadaveric specimens using a previously‐validated linear FEA method.^(^ ^20^ ^)^ Specifically, the linear FE modeling technique was directly applied to the μCT image using a voxel grid mesh model at the original isotropic image resolution of 28.5 μm. Binary bone voxels were determined using a segmentation method based on manually selected fixed thresholding and connectivity analysis.

### Optimization of nonlinear FEA parameters

Nonlinear FEA parameters were optimized using the first of the three repeat CT scans of each cadaveric specimen, and the modulus measures were computed over the cylindrical VOI (see VOI Selection) at the 4% to 6% tibial site. The substep number and the force and displacement convergence parameters of the FEA algorithm were experimentally optimized within the ANSYS setup. For this purpose, a “relative modulus” metric was defined for a given specimen and parameter setup as the percentage of the highest modulus value obtained for the specific specimen at any parameter setup. The optimum value for a specific parameter was selected to minimize computation complexity, while achieving the 100% relative modulus for all specimens. Optimum values of the three FEA parameters were determined in three sequential steps. First, the substep number was optimized over the list {5, 10, 20, 30, 40, 50, 100, 150, 200, 250} using the ANSYS default values for force and displacement tolerance parameters. (ANSYS default values for the substep number and tolerance for force and displacement convergence were 10, 0.5%, and 5%, respectively.) Next, the force tolerance parameter was optimized over 0.05%, 0.1%, 0.5%, and 1% using the optimum substep number and the default displacement tolerance parameter. Finally, the optimal value of the displacement tolerance parameter was decided over 0.5%, 1%, 5%, and 10% using the optimum substep number and force parameter.

**Fig. 5:**
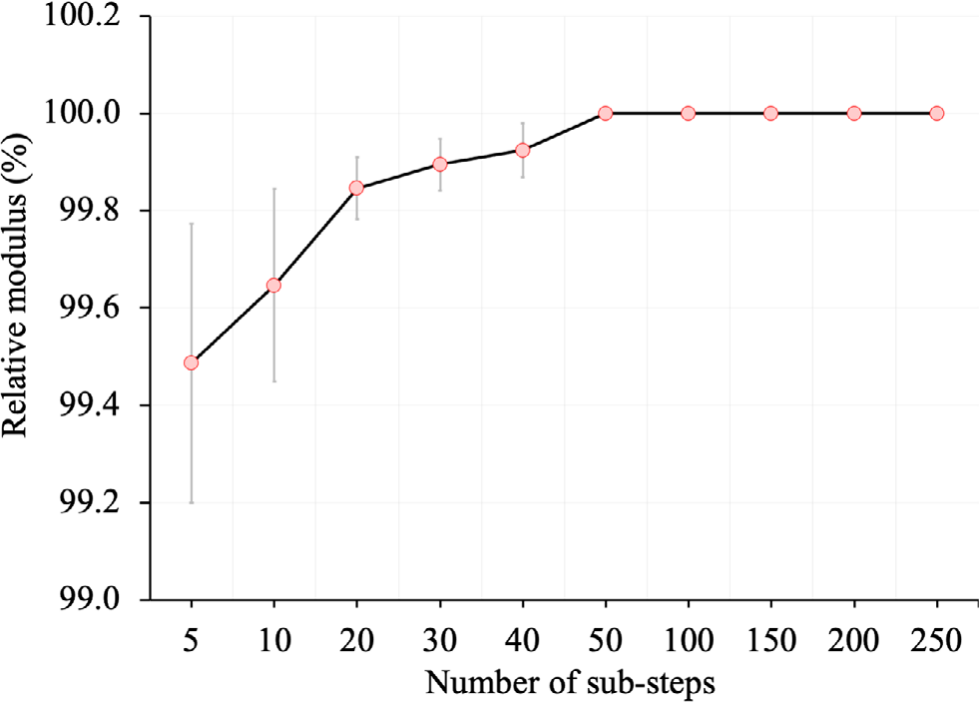
Substep optimization of FEA under compressive loading. Mean and standard deviation of relative modulus values are shown as a function of substep number. At substep number of 50 and higher, 100% relative modulus was achieved for all specimens. For both x‐ and y‐shear loading, 100% relative modulus was observed for all specimens at the smallest substep number of 5 (data not shown).

Fig. 5 plots the relative modulus performance at different substep parameters, which shows suboptimal performance or <100% relative modulus with high SD at lower values of the parameter. However, with the increase in the values of the substep parameter, the mean values of relative modulus were increased and the SD values were reduced; finally, the relative modulus value reached 100% for the substep number ≥50. For both shear moduli (x and y), 100% relative modulus with 0 SD was achieved for all values of the substep parameter. The maximum performance of 100% relative modulus was observed using the optimum substep number and for all combinations of force and displacement parameters. Therefore, the optimum value of substep number of 50 was chosen as the smallest step‐number yielding 100% relative modulus performance for all specimens. No effects of varying force and displacement parameters was observed on FEA results. Therefore, the ANSYS default values of 0.5% and 5% were used for force and displacement tolerance parameters, respectively, for all experiments presented in this work.

### Experiments and data analysis

Our experiments were designed to (i) evaluate its performance and (ii) apply the method to in vivo data. The performance of the method under compressive as well as shear loading was examined to evaluate: (i) correlation of CT‐based modulus measures with μCT‐derived reference values, (ii) repeat scan reproducibility, and (iii) stress propagation along bone microstructure. The in vivo experiment was designed to evaluate the association of clinical CT‐derived bone strength measures with sex, body size and composition measures.

Means and SDs of CT‐derived and μCT‐derived values of different modulus measures were computed, and the shift in values of different μCT‐derived and CT‐derived modulus measures was analyzed using the Bland‐Altman plot. The association of CT‐derived values of a modulus measure with the μCT‐derived reference values was evaluated in terms of Pearson correlation coefficient (*r* value). Shapiro‐Wilk normality test^(^ ^43^ ^)^ of residuals was performed to examine the accuracy of fitted linear regression models between CT and μCT FEA measures. Also, linear correlations of μCT‐derived bone volume fraction (BV/TV) and CT‐derived BMD measures with μCT‐derived FE modulus measures were analyzed to examine the ability of ex vivo and in vivo density measures to predict bone strength.

For the reproducibility experiment, bone modulus measures were computed over cylindrical VOIs at the 4%–6% tibial site from three repeat CT scans. To capture variability of the entire process including the imaging and processing cascade, the VOIs of individual repeat scans were computed independently. Finally, the reproducibility of each of the different bone measures was computed in terms of intraclass correlation coefficient (ICC) of the measures derived from the repeat scans.

Stress propagation along the Tb microstructure and marrow space was evaluated using a stress histogram over the segmented Tb microstructure and marrow regions. These histograms were normalized so that the area under them was constant. Cylindrical VOIs at the 4%–6% tibial site from the first of the three repeat CT scans of cadaveric specimens were used for this experiment. Means and SDs of stress over the Tb microstructure and marrow regions were computed and unpaired t tests were performed. Segmentations of Tb microstructure and marrow regions were generated using a previously‐validated algorithm.^(^ ^44^ ^)^ The algorithm is based on automated assessment of space varying marrow intensity and bone‐marrow contrast, thresholding, and connectivity analysis. It may be clarified that binary separation of Tb and marrow regions was required only for the evaluative purpose and was not used for FEA.

For the in vivo experiment, the axial VOI with 50% peel over the 4%–6% tibial site was used. Means and SDs of different modulus measures among males and females were computed to describe their normative distributions. Pure lean mass (kg) was estimated from DXA whole body (WB) scans by subtracting the WB bone mineral content from the WB nonfat mass, and the lean tissue mass index (LTMI) (kg/m^2^) was computed as the ratio of (pure lean mass in kg)/(height in m)^2^. Pearson correlation analysis was performed to determine the associations of different modulus measures with height (cm), weight (kg), body mass index (BMI) (kg/m^2^), and lean mass. Shapiro‐Wilk normality tests^(^ ^43^ ^)^ were performed to examine normality of the distributions. Unpaired *t* tests were performed to examine the differences in bone modulus measures for males and females. Also, the group differences in different measures were analyzed in terms of unadjusted and adjusted effect sizes. Unadjusted effect size was calculated as (difference of the group means)/(pooled SD), whereas the adjusted effect size was calculated as (difference of the least square means of the groups)/√MSE, where MSE is the mean square error from the general linear model that included pure lean mass as covariates.

## Results

**Fig. 6:**
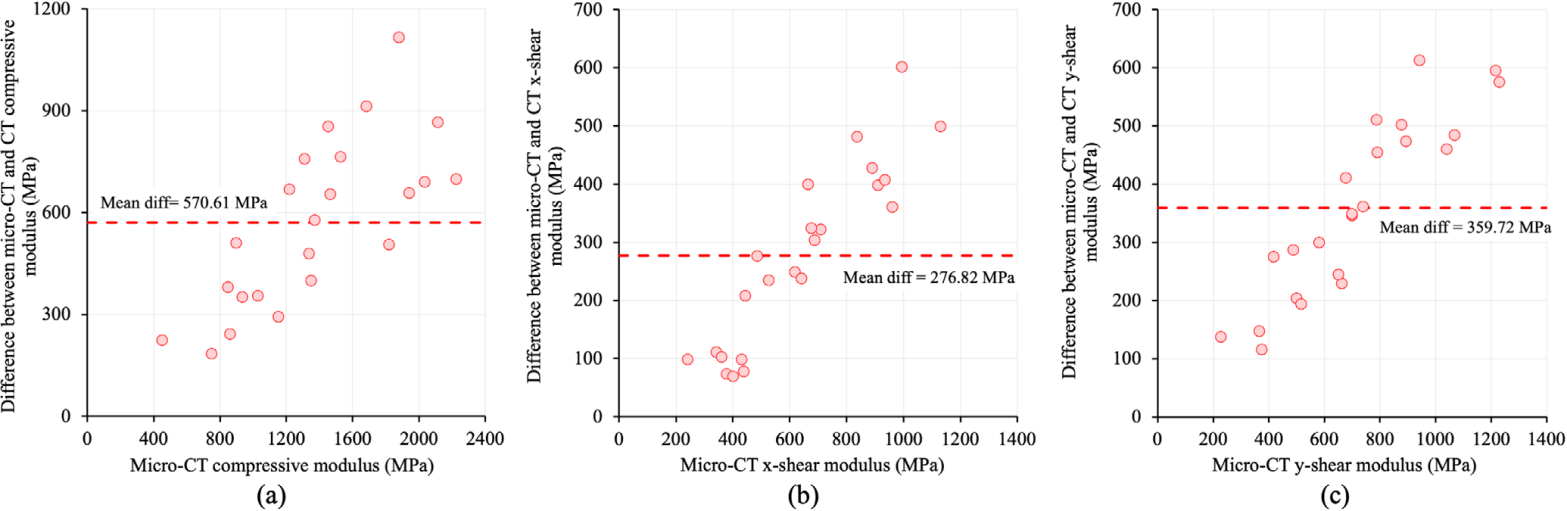
Bland‐Altman plot of differences between CT‐derived and μCT‐derived values of compressive (*A*), x‐shear (*B*), and y‐shear (*C*) moduli, respectively.

**Fig. 7:**
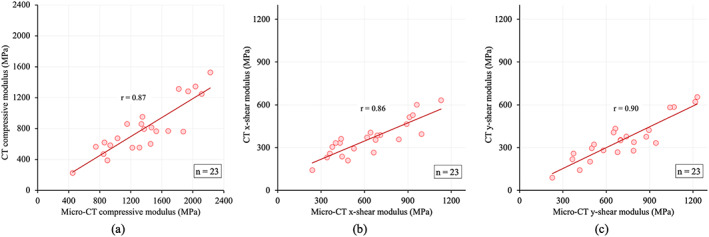
Linear correlation of CT‐based compressive (*A*), x‐shear (*B*), and y‐shear (*C*) modulus measures with μCT‐derived reference values, respectively.

Summary statistics (mean ± SD) and linear correlation of different Tb modulus values from CT and μCT images are shown in Table 1. Mean ± SD of CT‐derived compressive, x‐ and y‐shear modulus were 805.22 ± 335.49, 362.44 ± 122.33, and 355.07 ± 146.17 MPa, respectively, and those values for μCT were 1375.83 ± 477.42, 639.28 ± 247.99, and 714.80 ± 269.63 MPa, respectively. Fig. 6*A‐C* shows the Bland‐Altman plot of the difference between μCT‐derived and CT‐derived modulus values. The mean difference between μCT‐derived and CT‐derived compressive, x‐ and y‐shear modulus values were 570.61, 276.82, and 359.72 MPa. Linear correlation of 0.87, 0.86, and 0.90 were observed (*n* = 23) between CT‐derived and μCT‐derived values of compressive, x‐ and y‐shear modulus, respectively (Fig. 7*A‐C*). For all three modulus measures, residuals were computed from the linear regression line and were found to be normally distributed with *p* values of Shapiro‐Wilk normality test for compressive, x‐ and y‐shear modulus being 0.27, 0.48, and 0.63, respectively. Results of intramodality and intermodality correlation analysis between bone density and mechanical measures are presented in Table 2. μCT‐derived BV/TV measure showed higher linear correlations (*r* ∈ [0.95 0.99]) with μCT‐derived modulus measures than the CT‐derived BMD values (*r* ∈ [0.74 0.81]). Also, repeat CT scan ICC values of 0.97, 0.97, and 0.98 were observed (*n* = 24) for FEA‐derived compressive, x‐ and y‐shear modulus, respectively.

**Table 1:** Summary Statistics (mean ± SD) of CT‐Derived and μCT‐Derived Tb Measures and their Linear Correlations

Modulus (MPa)	CT (mean ± SD)	μCT (mean ± SD)	*r*	Calibration^a^	
				Slope	Intercept
Compressive (MPa)	805.22 ± 335.49	1375.83 ± 477.42	0.87	1.24	375.90
X‐shear (MPa)	362.44 ± 122.33	639.28 ± 247.99	0.86	1.75	6.24
Y‐shear (MPa)	355.07 ± 146.17	714.80 ± 269.63	0.90	1.65	126.02

^a^ CT modulus values were calibrated into μCT‐derived reference values.

**Table 2:** Intramodality and Intermodality Correlations Between Tb Density and Modulus Measures

	μCT‐derived modulus measures		
Measures (modality)	Compressive (MPa)	X‐shear (MPa)	Y‐shear (MPa)
BV/TV (μCT)	0.99	0.95	0.96
BMD (CT)	0.81	0.74	0.77

**Fig. 8:**
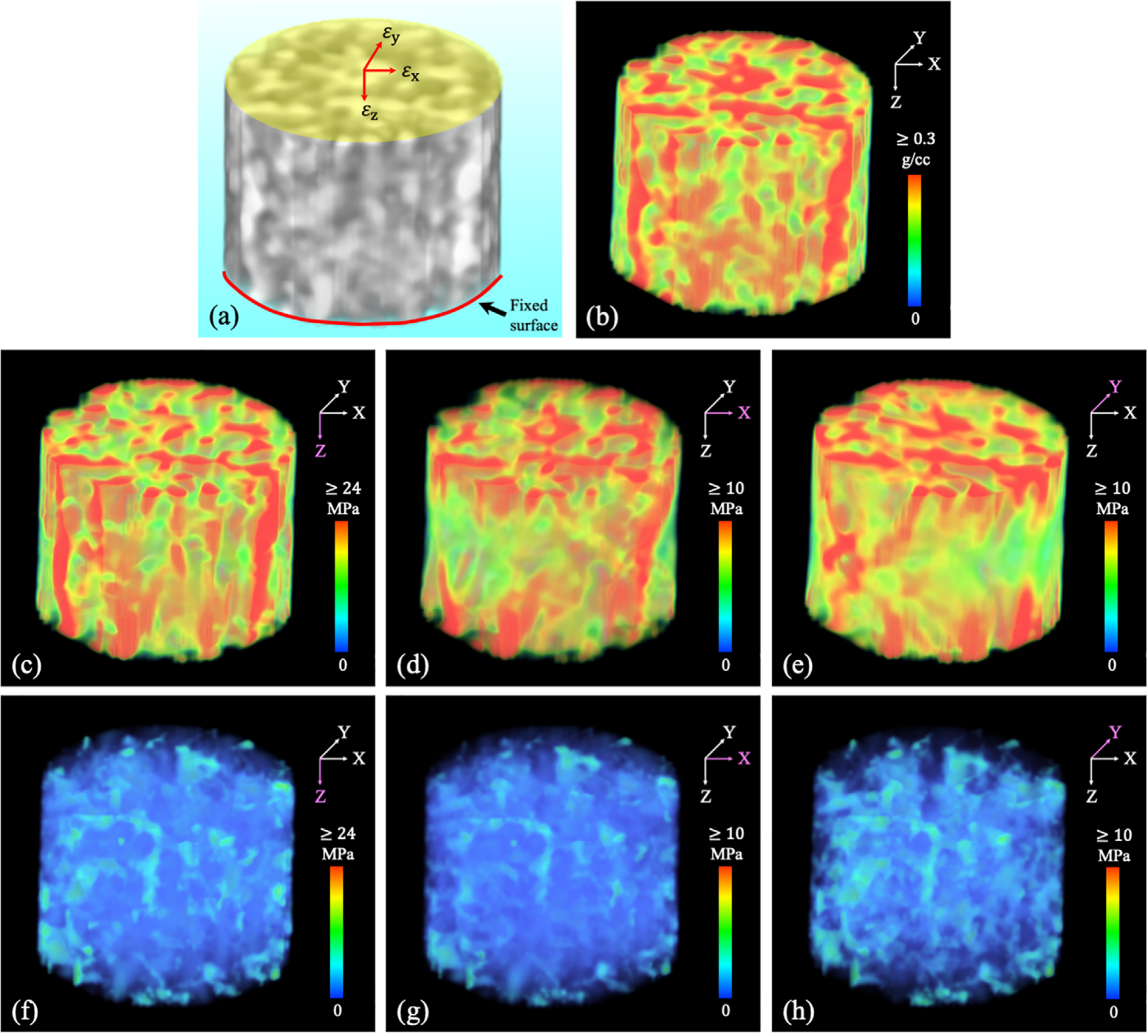
CT‐based FEA of Tb microstructure at different loading conditions. (*A*) Boundary and loading conditions for FEA on a cylindrical Tb core. (*B*–*E*) Color‐coded volume rendition of CHA density distribution (*B*), von Mises stress distribution under compressive (*C*), and x‐ and y‐ shear (*D*,*E*) loading conditions. (*F*–*H*) Stress leakages over marrow space under different loading conditions. Maximum stress value of 24 MPa is used for compressive stress distributions (*C*,*F*), whereas 10 MPa is used for shear stress distributions (*D*,*E*,*G*,*H*).

**Fig. 9:**
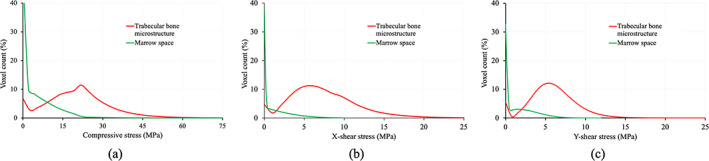
Normalized histogram of stress distribution over segmented Tb and marrow microregions for compressive (*A*), x‐shear (*B*), and y‐shear (*C*) loading conditions, respectively.

Experimental results examining stress‐flow along Tb microstructure, the fundamental principle of structural mechanics, at in vivo CT‐based nonlinear FEA are presented in Figs. 8 and 9. Fig. 8 shows qualitative results of the FEA‐computed stress distribution over a cylindrical VOI. Specifically, Fig. 8*A* shows the volume rendition of a cylindrical VOI at 4%–6% tibia, loading surface where the displacement was applied, and the bottom fixed surface. Fig. 8*B* presents color‐coded volume rendition of the CHA density map over the target VOI. Fig. 8*C*–*E* shows the von Mises stress distributions under different loading conditions. Fig. 8*F*–*H* shows volume rendition of von Mises stress over the segmented marrow region using the same color‐scale as that illustrates stress leakage in marrow. Fig. *A‐C* illustrates normalized stress histograms over the segmented Tb (red) and marrow (green) regions under compressive, x‐ and y‐shear loading conditions. These histograms were computed over all VOIs (*n* = 3*24) from cadaveric CT scans used for reproducibility experiments. For any loading condition, the histogram representing the stress distribution over marrow region shows that most marrow voxels absorbed no stress, and the count suffering nonzero stress falls rapidly. Mean ± SD of stresses supported by marrow voxels were 4.11 ± 5.30, 1.24 ± 1.09, and 1.43 ± 1.86 MPa under compressive, x‐ and y‐shear loading conditions, respectively. Under any loading condition, the Tb stress histogram mode was found at a higher value than the average marrow stress under the same loading condition. Mean ± SD of stress over the Tb micro‐network were 22.31 ± 12.21, 7.12 ± 2.52, and 7.86 ± 4.38 MPa under compressive, x‐ and y‐shear loading conditions, respectively. The differences in stress distributions over the Tb and marrow regions were statistically significant (*p* < 1 × 10^−11^).

Table 3 summarizes the statistics of height, weight, pure lean mass, BMI, and LTMI for male (*n* = 50) and female (*n* = 50) subjects of our in vivo study. Height, weight, pure lean mass, and LTMI for males were significantly higher (*p* < 0.01) than the females, whereas the group difference for BMI was not significant. Large effect sizes (>0.8) between males and females were observed for height, weight, pure lean mass, and LTMI, whereas that for BMI was small (<0.5). The highest effect size of 2.23 was observed for pure lean mass.

**Table 3:** Characteristics of IBDS Sample for in Vivo Analysis and Comparison Between Male and Female Participants

Attributes	Males (*n* = 50) (mean ± SD)	Females (*n* = 50) (mean ± SD)	*t* test (*p*)	Effect size^a^
Age at scan	19.80 ± 0.70	19.80 ± 0.70	0.83	−0.04
Height (cm)	179.42 **±** 8.24	167.35 **±** 6.64	<0.01	1.61
Weight (kg)	85.38 **±** 16.62	69.06 **±** 17.94	<0.01	0.94
Pure lean mass^b^ (kg)	63.91 **±** 10.07	44.25 **±** 7.35	<0.01	2.23
BMI^c^ (kg/m^c^)	26.50 **±** 4.79	24.54 **±** 5.72	>0.05	0.37
LTMI^d^ (kg/m^c^)	19.83 **±** 2.70	15.74 **±** 2.09	<0.01	1.69

^a^ Effect size was calculated as (mean difference)/(pooled SD).

^b^ Pure lean mass is estimated from DXA whole‐body (WB) scan as (WB non‐fat mass) − (WB bone mineral content).

^c^ BMI (body mass index) = weight/height^2^.

^d^ LTMI (lean tissue mass index) = pure lean mass/height^2^.

Comparative results of the different modulus measures for males and females are shown in Table 4. All three moduli from males and females together were found to be normally distributed, where *p* values of Shapiro‐Wilk normality test for compressive, x‐ and y‐shear modulus were 0.32, 0.25, and 0.18, respectively. Modulus values for males under all three loading conditions were significantly higher (*p* < 0.01) than the values for females under matching loading conditions. The unadjusted effect size between the two groups was high (>0.8) for compressive modulus, while effect sizes for both x‐ and y‐shear moduli were moderate (>0.5). However, effect sizes for different moduli were reduced to small (<0.5) or negligible (<0.2) values after adjusting for pure lean mass. Results of linear correlation analysis (*n* = 100) between different modulus measures and different attributes are presented in Table 5. LTMI and pure lean mass showed the highest correlations with different modulus measures, and higher values of LTMI and pure lean mass were associated with stronger bones. Compared to LTMI and pure lean mass, body weight and BMI showed relatively lower correlations with bone modulus, whereas height showed the smallest correlation.

**Table 4:** Comparisons of CT‐Based FEA‐Derived Modulus Measures Between Male and Female Participants

Modulus (MPa)	Males (*n* = 50) (mean ± SD)	Females (*n* = 50) (mean ± SD)	*t* test (*p*)	Effect size^a^	
				Unadjusted	Adjusted
Compressive	994.13 **±** 233.69	785.11 **±** 232.84	<0.01	0.90	0.05
x‐shear	564.79 **±** 128.45	496.70 **±** 121.04	<0.01	0.55	0.37
y‐shear	494.03 **±** 125.86	418.79 **±** 118.03	<0.01	2.62	0.28

^a^ Unadjusted effect size was calculated as (Difference of the group means)/(pooled std). Adjusted effect size was calculated as the (Difference of the least square means of the groups)/ √ MSE, where MSE is the mean square error from the general linear model that included pure lean mass as covariates.

**Table 5:** Associations of CT‐Based Modulus Measures With Body Size and Body Composition

Attributes	Compressive modulus (MPa)	x‐shear modulus (MPa)	y‐shear modulus (MPa)
Height (cm)	0.39	0.25	0.28
Weight (kg)	0.51	0.43	0.45
Pure lean mass (kg)	0.56	0.45	0.47
BMI (kg/m^2^)	0.41	0.39	0.39
LTMI (kg/m^2^)	0.56	0.49	0.49

Pearson correlation coefficients are reported.

All cylindrical VOIs for cadaveric experiments were of fixed size with the numbers of vertices, edges, and volume elements being 97695, 288574, and 93236, respectively, and there were 2171 vertices on the loading surface. Mean ± SD of the runtime for nonlinear FEA under compressive and shear loading were 13.23 ± 1.74 and 10.32 ± 3.03 minutes, respectively. For the in vivo experiments, vertex element counts on the loading surface were 11447 ± 1636, and vertex, edge, and volume element count over the VOI were 615572 ± 67053, 1827355 ± 204442, and 596350 ± 68698, respectively. Runtime for CT‐based FEA for the in vivo experiment was 65.31 ± 10.26 minutes.

## Discussion

High linear correlation was observed between CT‐derived and μCT‐derived FE modulus measures for both compressive and shear loading conditions. It was observed in the Bland‐Altman plot that CT‐based FEA underestimates the values of all three modulus measures compared to the μCT‐derived reference values. Moreover, a linear trend was observed in the difference between the CT‐derived and μCT‐derived modulus values. Although, the CT‐based modulus values were lower than the μCT‐derived reference values, high linear correlations among them suggest that the CT‐based nonlinear FEA provides reliable surrogate modulus measures of Tb microstructure at in vivo imaging, where voxel resolution is comparable or nominally lower than individual trabecular thickness. The linearity of the association between CT‐derived and μCT‐derived modulus values was confirmed by Shapiro‐Wilk normality test that showed the residuals computed from the linear regression line to be normally distributed for all three modulus measures.

Several HR‐pQCT‐based studies have reported higher correlation (*r* ^2^ > 0.9) between HR‐pQCT and reference μCT‐derived mechanical measures.^(^ ^45^, ^46^, ^47^, ^48^, ^49^ ^)^ However, those findings are based on studies performed on small ex vitro specimens with or without marrow. MacNeil and Boyd^(^ ^50^ ^)^ observed a linear correlation of *r* = 0.85 in their study comparing FEA measures derived from cadaveric HR‐pQCT imaging acquired under an in vivo condition with μCT‐derived reference values. It may be noted that the linear correlation of different modulus measures (*r* ∈ [0.86 0.89]) observed in the current study between CT imaging under in vivo condition and ex vivo μCT imaging is similar to the correlation observed by MacNeil and Boyd^(^ ^50^ ^)^ in their HR‐pQCT‐based study. Additionally, it may be clarified that the performance observed in the current study was obtained using a whole‐body clinical CT scanner.

It may also be clarified that the true resolution (0.3 mm) of the clinical CT scanner used in this study was sufficient for capturing marrow voids. However, CT resolution was higher than the average thickness (~0.15 mm) of human Tb, and binary segmentation of the Tb micro‐network from CT images often creates discontinuity along individual trabeculae leading to disruption in FEA stress flow along the Tb micro‐network. To the best of our knowledge, the validity of nonlinear FEA to compute bone strength using clinical CT imaging at an in vivo condition without requiring binary segmentation of Tb micro‐network, whereas accounting for bone micro‐distribution at the level of individual trabeculae was not examined earlier. This study validates a method that will deliver a unique tool useful in research and clinical human studies exploring impacts of diseases or therapeutic interventions on bone microstructural strength and fragility. Most modern clinical CT scanners provide similar or better spatial image resolution and faster scanning, and the FEA method may be readily replicated in on such clinical CT scanners.

It is worth mentioning that previous studies have demonstrated that BMD correlates with strength measures.^(^ ^51^, ^52^, ^53^, ^54^ ^)^ Specifically, high correlations were observed when both BMD and FE modulus measures were computed from the same images.^(^ ^53^, ^54^ ^)^ The goal of the current study was to examine the ability of clinical CT‐derived FE modulus measures to predict ex vivo μCT‐derived FE modulus measures, which are known to highly correlate with experimental measures. As observed in the intramodality and intermodality comparison experiment, bone density highly correlates with modulus measures when both measures are computed from the same imaging modality. Clinical CT‐derived BMD measures showed a relatively low correlation with the μCT‐derived FE modulus measures as compared to the correlation between CT‐derived and μCT‐derived FE modulus measures. Thus, clinical CT‐derived FE measures add values beyond the BMD measure in predicting the bone strength captured by ex vivo imaging. Also, high repeat scan reproducibility was observed among the FEA‐derived modulus values, suggesting that the entire process including imaging, preprocessing, and nonlinear FEA is reproducible and suitable for multicenter longitudinal studies.

Our experimental results suggest that stress‐flow is primarily confined within Tb microstructures, complying with the fundamental principle of structural biomechanics at resolutions acheivable by clinical CT imaging. It was qualitatively observed in Fig. 8*C* that with nonlinear FEA and compressive loading conditions, high stress lines (red) follow Tb micro‐structures (red). Only low stress values appearing in blue colors (Fig. 8*F*) were observed over the marrow region implying minimal stress leakage through the marrow space. Similar results were observed for von Mises stress distributions under shear loading conditions. It is worth mentioning that high stress primarily flows along the loading direction and may not represent the entire Tb network. Instead, it stands for a subset of Tb voxels supporting the stress flow through the network. This phenomenon was more prominent under shear loading conditions. These observations were confirmed by quantitative analysis showing that stresses experienced by most Tb voxels were higher than the average stress over the marrow region. These observations suggest that modulus measures derived from clinical CT imaging using a nonlinear FEA describe mechanical properties of the Tb micro‐network without requiring binary separation between Tb and marrow voxels. To the best of our knowledge, the relationships between stress distribution and Tb microstructure has not been studied or demonstrated in previously reported studies related to nonlinear FEA on bone,^(^ ^21^, ^22^, ^24^, ^25^, ^27^ ^)^ and these methods did not aim to characterize stiffness of Tb microstructure. The relaxation of binary separation between Tb and marrow, demonstrated in this work, will expand the scope of bone micro‐FEA at in vivo imaging, where Tb micro‐network appearance is fuzzy and binary segmentations are challenging and prone to errors.

The goal of our in vivo human study was to examine the associations of different modulus measures with sex, body size, and composition metrics. Our observation that males are associated with higher modulus values or stronger bones than females is consistent with ex vivo results reported on cadaveric studies using HR‐pQCT imaging and mechanical experiments.^(^ ^13^, ^14^ ^)^ Reduced effect sizes in all three moduli values after adjusting for pure lean mass suggest that much of the sex‐related difference in bone stiffness are mostly explained by differences in body composition between males and females. This finding is consistent with the observation in Table 3 that males have much more lean mass than females and the observation in Table 5 that bone modulus measures have high correlation with lean mass. These observations are further supported by our finding that BMD is positively associated with bone stiffness and those reported in DXA‐based human studies^(^ ^16^, ^55^, ^56^ ^)^ that lean mass has a stronger association with BMD than BMI and other body‐size measures. Also, these findings add to the explanation of the observation in a study involving men and women aged ≥40 years that loss of lean mass increases fracture risk independent of other factors.^(^ ^57^ ^)^ Among the three FE measures, compressive modulus showed stronger associations with various body size and composition attributes than different shear moduli. A speculative justification behind this observation may be that compressive loading is aligned with the body weight‐bearing direction, thus, receiving more biomechanical signals and aligning bone microstructures with body size and composition attributes. The novelty of our observation is that our experimental results confirm a strong association between pure lean mass and CT‐derived modulus measures, and this finding aligns with epidemiological and clinical reports showing the importance of lean mass to strong bone and provides support for public health and clinical fracture programming that emphasis the acquisition and preservation of lean tissue.^(^ ^57^ ^)^ Finally, the results of our in vivo human study are novel because it establishes the feasibility of the application of FEA in in vivo studies, where acquired scans are often associated with motion and other artifacts.

It is worth mentioning that, due to high computational complexity at μCT resolution, we used small VOIs for validation experiments. On the other hand, for in vivo experiments, VOIs were defined using 50% peel and the reference of tibial length to account for varying bone length and width among participants. VOIs with larger widths include denser Tb microstructures closer to the cortical bone and thus associate with greater stiffness, and it may partially explain the differences in CT‐based average modulus values observed in our validation and in vivo experiments. In general, larger VOIs are associated with greater smoothing of noisy errors. Thus, a larger VOI is expected to produce similar or better correlation between computed Tb modulus values derived from CT and the reference μCT imaging. Also, there are a few limitations of the human study reported in this work. Participants were all white, limiting the generalizability of our findings to other races and ethnicities.^(^ ^58^ ^)^ Additionally, the IBDS represents a rural population, and the results may not be applicable to an urban population with a different lifestyle.^(^ ^59^ ^)^ Finally, the sample size of this human study is small, although findings are statistically significant.

## Author Contributions

**Indranil Guha:** Conceptualization; formal analysis; methodology; software; validation; visualization; writing – original draft; writing – review and editing. **Xialiou Zhang:** Methodology; software; validation; visualization; writing – review and editing. **Chamith Rajapakse:** Methodology; writing – review and editing. **Elena M. Letuchy:** Data curation; formal analysis; validation; writing – review and editing. **Gregory Chang:** Methodology; writing – review and editing. **Kathleen Janz:** Methodology; writing – review and editing. **James C. Torner:** Methodology; writing – review and editing. **Steven M. Levy:** Methodology; resources; writing – review and editing. **Punam K. Saha:** Conceptualization; funding acquisition; investigation; methodology; project administration; resources; software; supervision; validation; visualization; writing – original draft; writing – review and editing.

## Conflicts of Interest

All authors state that they have no conflicts of interest.

### Peer Review

The peer review history for this article is available at https://publons.com/publon/10.1002/jbm4.10627.

## Data Availability

The data that support the findings of this study are available from the corresponding author upon reasonable request.
